# Clinical Implications of Incorporating Molecular Profiles into the Staging of Endometrial Cancer: A Critical Review of the 2023 FIGO System on the Wave of 2025 ESGO/ESTRO/ESP Guidelines

**DOI:** 10.3390/cancers18172748

**Published:** 2026-08-24

**Authors:** Angela Santoro, Giuseppe Angelico, Antonio d’Amati, Livia Maccio, Emma Bragantini, Francesco Fanfani, Anna Fagotti, Gian Franco Zannoni

**Affiliations:** 1 Pathology Unit, Department of Laboratory and Hematology Sciences Fondazione Policlinico Universitario, Fondazione Policlinico Universitario Agostino Gemelli IRCCS, Largo A. Gemelli 8, 00168 Rome, Italy; angela.santoro@policlinicogemelli.it (A.S.); damatiantonio@yahoo.it (A.d.); 2 Pathology Institute, Catholic University of Sacred Heart, 00168 Rome, Italy; 3 Department of Medicine and Surgery, Kore University of Enna, 94100 Enna, Italy; giuangel86@hotmail.it; 4 Anatomic Pathology, Department of Translational Medicine, University of Ferrara, 44121 Ferrara, Italy; livia.maccio@unife.it; 5 Department of Pathology, Ospedale San Maurizio, 39100 Bolzano, Italy; emma.bragantini@sabes.it; 6 Gynecologic Oncology Unit, Department of Women, Children and Public Health Sciences, Fondazione Policlinico Universitario Agostino Gemelli IRCCS, 00168 Rome, Italy; francesco.fanfani@unicatt.it (F.F.); anna.fagotti@unicatt.it (A.F.); 7 Catholic University of the Sacred Heart, 00168 Rome, Italy

**Keywords:** endometrial carcinoma, TCGA, serous carcinoma, clear cell carcinoma, undifferentiated carcinoma

## Abstract

This review examines the clinical and practical implications of embedding molecular profiles directly into the 2023 FIGO staging system for endometrial carcinoma, in the context of the 2025 ESGO/ESTRO/ESP guidelines. The primary purpose is to navigate a central conflict in modern oncology: how to deliver increasingly personalized care while maintaining a globally accessible, equitable, and standardized cancer classification system. The 2023 FIGO update represents a paradigm shift from the traditional dualistic model (Type I versus Type II) by allowing molecular findings to redefine stage itself. While this integration offers clear benefits, it introduces significant challenges. First, the system depends on advanced molecular testing, creating a “rich-poor” divide where patients in resource-limited settings are systematically overtreated because testing is unavailable. Second, stage becomes unstable, changing with sequential histologic and molecular re-review, which causes confusion for patients and clinicians. Third, the system lumps prognostically distinct histotypes (Serous, Clear Cell, Carcinosarcoma, and Grade 3 Endometrioid) into a single aggressive stage, obscuring meaningful differences in survival. Fourth, it relies on subjective parameters such as “substantial” lymphovascular space invasion, for which no standardized definition exists, leading to high inter-observer variability. After analyzing these controversies, the review proposes a pragmatic solution: decouple anatomical staging from molecular risk stratification. Staging should remain a purely anatomical, universally applicable descriptor of tumor extent, while molecular and histologic data are used separately within a dynamic risk assessment model, as suggested by the European guidelines. This dual-track approach preserves global comparability, reduces inequity, and maintains diagnostic stability, while still enabling personalized treatment where advanced diagnostics are available.

## Simple Summary

The present paper reviews the 2023 FIGO staging system for endometrial cancer, which for the first time incorporates molecular testing (like POLE mutations and p53 status) alongside traditional anatomical findings. This update reflects tumor biology and helps avoid over- or under-treating patients, though it also introduces some practical challenges. Access to molecular testing varies widely, so a patient’s cancer stage can depend partly on where they are treated, not just their tumor’s biology. Staging can also change after surgery once molecular results come back, which requires clear communication with patients. The paper also discusses inconsistent grouping of aggressive cancer subtypes and variability in measuring lymphovascular invasion as areas needing further standardization. We therefore propose that anatomical stage remain fixed and universal, while molecular and pathological findings guide a separate risk-stratification system for treatment planning, keeping staging simple and consistent worldwide while still enabling personalized care.

## 1. Introduction

For nearly four decades, the clinical management of endometrial carcinoma (ECs) was underpinned by the dualistic pathogenetic model proposed by Bokhman in 1983 [1]. This framework categorized tumors into two distinct types: Type I, comprising estrogen-dependent, low-grade endometrioid carcinomas associated with metabolic syndrome and a generally favorable prognosis; Type II, consisting of non-estrogen-dependent, high-grade, mostly serous carcinomas associated with an aggressive clinical course and poor outcomes [1]. While this binary system provided a necessary foundation for treatment planning, it increasingly failed to account for the biological heterogeneity observed in clinical practice [1,2]. A significant subset of clinically aggressive tumors exhibited “low-risk” histology, while some high-grade tumors behaved indolently, leading to both overtreatment and undertreatment [1,2].

The landscape of endometrial cancer classification underwent a significant shift with the publication of The Cancer Genome Atlas (TCGA) Research Network findings in 2013 [3]. By performing an integrated genomic, transcriptomic, and proteomic analysis, the TCGA identified four distinct molecular subgroups: POLE-ultramutated, Microsatellite Instability-Hypermutated (MSI-H), Copy-Number Low (CNL), and Copy-Number High (CNH) [3]. These molecular signatures were found to be superior predictors of clinical outcome compared to traditional histomorphology. This discovery necessitated the development of surrogate assays, primarily immunohistochemistry (IHC) and targeted sequencing, to translate these complex genomic findings into routine clinical practice, a process formalized by the ProMisE algorithm [3,4].

The 2023 FIGO staging system attempts to bridge the gap between this new biological understanding and anatomical staging. By incorporating molecular findings directly into the staging definitions, FIGO aims to create a system that reflects the true “nature” of the disease rather than just its extent [5]. However, this integration has sparked a significant debate within the medical community, which warns of potential “chaos” in clinical management [5,6]. The European Society of Gynaecological Oncology (ESGO), the European Society for Radiotherapy and Oncology (ESTRO), and the European Society of Pathology (ESP) have proposed a distinct “risk stratification” approach in their 2021 guidelines, where molecular data is integrated with anatomical staging to guide treatment, rather than altering the stage itself [4,7]. Following the new FIGO 2023 guidelines, in order to improve the quality of care for people with gynecological cancers, in 2025 ESGO, ESTRO, and ESP have updated the joint evidence-based guidelines in endometrial carcinoma, incorporating the revised 2023 FIGO staging [8].

Prognostic risks (low, intermediate, high intermediate, high, uncertain) are defined as estimated overall 5-year risk of recurrence in the low-risk group (<8%), intermediate-risk group (8–14%), high–intermediate-risk group (15–24%), and high-risk group (≥25%). Allocation to a prognostic risk group makes it necessary to know molecular classification in order to define the appropriate therapeutic management [8].

The aim of the present review is to conduct a comprehensive analysis of the clinical and practical implications of directly embedding molecular biology within the latest staging criteria for endometrial cancer. It seeks to navigate a critical conflict in modern oncology: the imperative to deliver ever-more precise, personalized care versus the fundamental need to maintain a globally accessible, equitable, and standardized system for cancer classification.

## 2. Literature Search Strategy

A literature search was performed to identify relevant evidence concerning the molecular classification of endometrial carcinoma, the 2023 FIGO staging system, and the 2025 ESGO/ESTRO/ESP recommendations. PubMed/MEDLINE and Scopus were searched for publications addressing endometrial carcinoma in relation to molecular classification, POLE mutations, mismatch repair deficiency, p53 abnormalities, NSMP, histological subtypes, lymphovascular space invasion, FIGO 2023 staging, stage migration, prognostic stratification, and treatment implications. The search included peer-reviewed original studies, systematic reviews, narrative reviews, international guidelines, consensus statements, and relevant large retrospective or prospective cohort studies. Particular emphasis was placed on recent literature published between 2021 and 2026, while seminal studies and earlier publications were retained when necessary to provide historical or biological context. Reference lists of relevant articles and guidelines were also manually screened to identify additional pertinent publications. Studies were considered eligible when they provided clinically, pathologically, or molecularly relevant information directly applicable to the objectives of this review. Articles unrelated to endometrial carcinoma or to the molecular, pathological, prognostic, or staging aspects discussed in the review were excluded. Given the narrative and critical nature of this review, the search was intended to provide a comprehensive and up-to-date evidence base rather than to perform a formal systematic review or quantitative meta-analysis.

## 3. The Molecular Landscape: From TCGA to Clinical Practice

The translation of the TCGA molecular classification into the clinical setting has fundamentally altered how clinicians perceive endometrial cancer risk. The four molecular subtypes identified (POLE-mutated, MMRd, p53-abnormal, and NSMP) carry distinct biological behaviors that often override traditional histological grading, necessitating a re-evaluation of therapeutic paradigms (Table 1) [4,9,10,11,12,13].

The POLE-mutated (POLEmut) subgroup, although the rarest (representing approximately 7–10% of cases), has the most profound clinical implications [4,9,10,11,12,13]. These tumors harbor pathogenic mutations in the exonuclease domain of the POLE gene, leading to an ultra-mutated phenotype and a robust anti-tumor immune response [4,9,10,11,12,13]. The most striking characteristic of POLEmut tumors is their exceptionally favorable prognosis, with 5-year recurrence-free survival exceeding 95%, a persistence noted even in the presence of traditional high-risk features such as high-grade histology, substantial lympho-vascular space invasion (LVSI), or older patient age.

Accordingly, the 2023 FIGO classification introduced Stage IAmPOLEmut to acknowledge the exceptionally favorable prognosis of these tumors [4,9,10,11,12,13]. This designation implies that patients with stage I or II disease who are POLE-mutated may safely undergo de-escalation of therapy, avoiding adjuvant chemotherapy or radiation that would otherwise be indicated by their histology, a principle under investigation in trials like PORTEC-4a [14,15]. This approach is the gold standard for care, but it has one important limitation: it depends entirely on advanced genetic testing. In resource-limited settings, the inability to identify these “super-survivors” results in systematic overtreatment, directly linking a patient’s therapeutic burden to the diagnostic capabilities of their institution.

Conversely, the p53-abnormal (p53abn) subgroup represents the most aggressive end of the spectrum [4,9,10,11,12,13]. Correlating with the TCGA “Copy-Number High” group, these tumors exhibit extensive somatic copy-number alterations and frequently harbor TP53 mutations [4,9,10,11,12,13]. While classically associated with serous carcinoma, p53 abnormalities are also found in a significant proportion of high-grade endometrioid carcinomas, carcinosarcomas, and some clear cell carcinomas [4,9,10,11,12,13]. The prognosis for p53abn tumors is poor, with high rates of recurrence and distant metastasis [4,9,10,11,12,13]. A critical finding is that approximately 50% of p53abn carcinomas exhibit Homologous Recombination Deficiency (HRD). This opens a therapeutic window for the use of PARP inhibitors, a strategy already well-established in ovarian cancer [16]. The 2023 FIGO system elevates the importance of this marker by using p53abn status as a key criterion for upstaging disease to Stage IIC or higher, mandating aggressive systemic therapy even for anatomically early-stage tumors [5,6]. Consequently, patients with p53-abnormal tumors are generally considered candidates for more intensive adjuvant treatment.

The Mismatch Repair Deficient (MMRd) subgroup, comprising about 25–30% of cases, occupies an intermediate prognostic position [17,18]. These tumors arise from the loss of DNA mismatch repair proteins (MLH1, PMS2, MSH2, MSH6), leading to microsatellite instability (MSI) [17,18]. A crucial insight is the gradient of MMRd prevalence; it is found in up to 40% of high-grade endometrioid carcinomas and is highly prevalent in undifferentiated carcinomas [4,9,10,11,12,13]. This challenges the historical axiom that high-grade histology uniformly signifies aggressive biology. Furthermore, the high mutational burden in MMRd tumors generates neoantigens that make them prime targets for immune checkpoint inhibitors, such as pembrolizumab, revolutionizing treatment for advanced disease [4,9,10,11,12,13]. While FIGO 2023 acknowledges MMRd, it does not accord it the same explicit staging power as POLE or p53 status [5,6]. In contrast, the ESGO guidelines leverage MMR status to stratify patients into intermediate-risk groups, potentially sparing them from the most intensive chemotherapy regimens (Table 2) [7]. This divergence highlights a key distinction: FIGO uses molecular data to redefine what the disease is (stage), while ESGO uses it to decide how to treat the disease (risk) [7].

Finally, the Non-Specific Molecular Profile (NSMP) subgroup remains the largest and most heterogeneous category. Often termed “Copy-Number Low,” these tumors lack POLE mutations, retain MMR protein expression, and have wild-type p53 [19,20]. For this group, which is typically composed of low-to-intermediate grade endometrioid carcinomas, molecular profiling offers less specific guidance [19,20]. Consequently, traditional clinicopathological parameters such as estrogen receptor (ER) status, progesterone receptor (PR) status, tumor grade, and LVSI retain their primary prognostic importance [19,20].

## 4. Histological Subtypes Re-Evaluated in the Molecular Era

Histology remains the cornerstone of endometrial carcinoma diagnosis, but its prognostic value is no longer sufficient on its own. Molecular classification has shown that tumors with similar morphology may behave very differently, making an integrated pathological assessment increasingly important [4,9,10,11,12,13]. However, as emphasized by the ESGO/ESTRO/ESP guidelines, molecular signatures frequently show poor correlation with traditional histological categories, creating a more complex biological map [7,8]. This discordance between a tumor’s appearance and its underlying biology complicates the diagnostic workflow and necessitates a move beyond treatment algorithms based solely on morphology, guiding us toward an integrated, multimodal classification model. The relationship between EC histotype and molecular profile has been summarized in Table 3.

### 4.1. Endometrioid Carcinoma (EEC)

Endometrioid carcinoma, the most common histotype, perfectly illustrates the molecular heterogeneity within a single diagnostic category [4,9,10,11,12,13]. While low-grade (G1–2) EECs are generally indolent and most often fall into the NSMP or MMRd categories, high-grade (G3) EEC presents a significant diagnostic and therapeutic dilemma [21,22]. Historically treated as a high-risk entity, molecular analysis reveals that G3 EEC is a mixture of all four TCGA molecular subtypes, with a significant proportion being MMRd or, less commonly, POLE-mutated [4,9,10,11,12,13]. The ESGO guidelines deliberately separate G3 EEC from non-endometrioid types in risk stratification, acknowledging this diversity [7,8]. In stark contrast, the 2023 FIGO system’s decision to group G3 EEC into the “aggressive” Stage IIC category (unless molecularly proven otherwise) risks substantial overtreatment for patients with molecularly favorable MMRd or POLEmut tumors, particularly where testing is unavailable [5,6]. Furthermore, within the NSMP group of EECs, additional biomarkers like CTNNB1 mutations (associated with worse outcome) and L1CAM overexpression (associated with poor prognosis similar to p53abn) are emerging as crucial refinements for risk stratification, suggesting that even this “non-specific” group requires further molecular dissection [23,24]. It is also recommended to test estrogen receptor status by immunohistochemistry in all endometrial carcinomas because it can help in diagnosis; it is considered prognostic in the NSMP group, and it is predictive for response to endocrine therapy in advanced and recurrent disease [7,8]. In this way, NSMP can be further classified as: (1) NSMP low-grade and estrogen receptor-positive (≥10%); (2) NSMP high-grade or estrogen receptor-negative (<10%) [25].

### 4.2. Serous Carcinoma (SC)

Serous carcinoma is the prototypical p53-abnormal tumor, with over 90% harboring TP53 mutations [26]. However, its diagnosis is not always straightforward. Morphological mimics exist, and critically, studies suggest that up to 20% of tumors diagnosed as high-grade serous based on morphology may be POLE-mutated or MMRd [27,28,29]. These molecularly favorable “mimics” can exhibit serous-like features with striking nuclear atypia. If treated based on morphology alone as aggressive serous carcinoma, these patients would be subjected to unnecessary, intensive therapy [27,28,29].

These observations support the routine incorporation of molecular testing into the diagnostic work-up of high-grade endometrial carcinomas. Moreover, the correct definition of a serous histotype and/or p53abn endometrial carcinomas can allow patients to be allocated to molecularly driven and biomarker-driven clinical trials [7,8].

Additionally, in advanced and recurrent settings, these types of cancers might be tested for HER2 overexpression by immunohistochemistry and, in case of an equivocal immunoreactive score, by in situ hybridization using standardized criteria, to address the ‘right’ patient to the anti-HER2 target therapy [30].

### 4.3. Clear Cell Carcinoma (CCC)

Traditionally grouped with serous carcinoma as a “Type II” tumor, clear cell carcinoma is molecularly distinct [4,9,10,11,12,13]. It is rarely p53-abnormal; instead, it most frequently falls into the NSMP or MMRd categories [4,9,10,11,12,13]. Clinically, its 5-year progression-free survival (~72%) is notably better than that of carcinosarcoma (~52%) or serous carcinoma, positioning it with an intermediate risk profile. Despite this, the 2023 FIGO system groups CCC into the same high-risk Stage IIC bucket as more aggressive types [5,6]. This categorization obscures its distinct biology, often involving ARID1A and PIK3CA mutations, and its intermediate prognosis [4,9,10,11,12,13]. The ESGO/ESTRO/ESP guidelines explicitly note that the behavior of MMRd and NSMP CCC is undefined, highlighting a key knowledge gap rather than assuming uniform aggressiveness [7,8].

### 4.4. Undifferentiated/Dedifferentiated Carcinoma (UEC/DEC) and Carcinosarcoma (UCS)

These high-grade tumors exemplify the critical role of molecular classification in seemingly unambiguous aggressive histotypes.

- Uterine Carcinosarcoma (UCS): Now classified as a metaplastic carcinoma, UCS is predominantly p53-abnormal and carries a very poor prognosis, potentially worse than serous carcinoma [31,32]. However, it is molecularly heterogeneous, with rare POLEmut cases showing excellent prognosis and a variable proportion (3–40% across studies) being MMRd [31,32]. Evidence suggests MMRd UCS may have a better prognosis, emphasizing the need for molecular testing even in this aggressive entity [31,32].
- Undifferentiated/Dedifferentiated Carcinoma (UEC/DEC): This highly aggressive tumor shows a molecular distribution similar to G3 EEC, with approximately half of cases being MMRd. While POLEmut UEC/DEC has an excellent prognosis, the prognosis of other subtypes is generally poor [33,34]. A critical refinement is the identification of SWI/SNF complex deficiency (e.g., loss of ARID1B, SMARCA4, SMARCB1), which is associated with an exceptionally poor prognosis, worse than conventional carcinosarcoma [33,34]. This finding suggests that within this already high-risk group, molecular profiling can identify a subset requiring the most aggressive management.

### 4.5. Rare Histotypes

Advances in molecular pathology have revealed rare endometrial carcinoma subtypes whose biological behavior cannot be adequately captured by the traditional dichotomous classification of “aggressive” versus “non-aggressive” disease, exposing the limitations of rigid staging systems. A practical algorithm for managing these entities is proposed in Table 4.

#### 4.5.1. Mesonephric-like Adenocarcinoma (MLEC)

MLEC is an aggressive tumor often misdiagnosed as endometrioid or clear cell carcinoma [35]. It is characterized by KRAS mutations (~70–80% of cases) and frequent ARID1A alterations, while typically showing wild-type p53 (NSMP) and lacking hormone receptor expression. The KRAS-MAPK signaling pathway is the primary driver, with recurrent mutations in PIK3CA and PTEN as secondary events. Clinically, MLEC exhibits a 5-year recurrence rate exceeding 50%, justifying its inclusion in high-risk categories regardless of stage. Importantly, the presence of KRAS mutations may offer therapeutic opportunities with MEK inhibitors in advanced or recurrent settings, though this remains investigational [35].

#### 4.5.2. Gastric-Type Carcinoma (GTEC)

GTEC is a deceptively bland but highly aggressive mucinous carcinoma that is hormone receptor negative and chemo-resistant [36]. Its molecular profile is distinct from standard TCGA categories, frequently harboring TP53 mutations (40–60%) but with low copy number alterations, placing most cases in a p53 abnormal/NSMP hybrid group [36]. Up to 30% of GTECs show ERBB2 (HER2) amplification, which may be targetable with anti-HER2 therapy, and KRAS/NRAS mutations occur in a minority [36]. GTEC exhibits a marked propensity for peritoneal and distant spread, with a 5-year disease-specific survival below 40%. Its bland morphology belies aggressive behavior, making expert pathologic review essential for accurate diagnosis [36].

#### 4.5.3. Neuroendocrine Carcinoma (NEEC)

NEEC shows molecular heterogeneity across all four TCGA groups. Pure small cell NEEC is typically p53-abnormal, highly aggressive, and frequently shows RB1 loss, with a 5-year survival under 30% [4,37]. In contrast, MMRd NEECs are often admixed with an endometrioid component and may have a more favorable prognosis, potentially benefiting from immune checkpoint inhibition [4,37]. Comprehensive molecular testing is therefore essential to guide therapy, as p53-abnormal NEEC may be candidates for platinum-based regimens combined with PARP inhibitors, while MMRd cases warrant immunotherapy consideration [4,37].

Another issue is represented by the emerging, rare, and ultra-rare histological variants of EC, such as giant cell carcinoma, endometrial carcinoma with histiocyte-like tumor cells, AFP-producing endometrial carcinoma, pilomatrix-like high-grade endometrioid carcinoma, corded and hyalinized endometrioid carcinoma, sertoliform endometrioid carcinoma, endometrial carcinoma with melanocytic differentiation, squamous endometrial carcinoma [38]. Considering the rarity of these variants and the frequent lack of appropriate follow-up information, time will be needed to fully understand their biologic behavior [38]. At the state of the art, the 2023 FIGO staging system does not consider these histological variants, and it would be inappropriate to include them a priori in the group of aggressive histological types, only because they display a histological appearance that is different from the low-grade endometrioid type [38]. Moreover, less is known regarding the relationships between these variants and other pathological parameters (myometrial invasion and LVSI) or molecular groups. The rigid categorization of the 2023 FIGO system struggles to accommodate the nuances of these rare and ultra-rare entities. Their management requires a flexible framework that integrates detailed histology with molecular insights, emphasizing the need for expert pathologic review and a personalized diagnostic approach beyond broad staging buckets [5,6].

## 5. The 2023 FIGO Staging System: Clinical Controversies

While the scientific rationale for the 2023 FIGO update is robust, its practical application has generated significant controversy, centering on its complexity, potential for inequity, and introduction of diagnostic instability [5,6].

The most pressing ethical and clinical concern is the “Rich and Poor” divide. The 2023 system’s accuracy is predicated on advanced molecular testing capabilities. In high-resource settings, these tests enable precise staging, such as downstaging a high-grade tumor to Stage IAmPOLEmut [5,6]. In resource-limited settings lacking such infrastructure, clinicians must default to staging based on histology alone. This creates a two-tiered system where a patient’s diagnosis and treatment depend more on their location and resources than on the biology of their tumor. A woman in a low-resource setting with a POLE-mutated tumor would be staged as IIC (aggressive) and receive chemotherapy, while her counterpart in a high-resource setting with the identical tumor would be staged as IAm and observed. This disparity may compromise the FIGO mandate that a staging system must be “comparable” across populations to allow for meaningful global benchmarking of cancer outcomes and resource allocation.

The incorporation of molecular findings into endometrial cancer staging also requires standardized and quality-controlled laboratory procedures. Pre-analytical factors, including tissue adequacy, fixation, tumor cellularity, and nucleic-acid quality, as well as analytical variables such as assay design, sequencing quality, validation, and variant interpretation, may affect the reliability of POLE and TP53 assessment. Pathology laboratories should therefore follow internationally recognized quality standards and use validated or verified assays with appropriate internal quality controls and, where available, external quality assessment. This is particularly important because technical errors or inter-laboratory variability may directly affect molecular classification, risk stratification, and, ultimately, FIGO stage.

Furthermore, the integration of molecular markers introduces the problematic phenomenon of Stage Migration and Diagnostic Instability [39]. Traditionally, cancer stage was a relatively fixed anatomical fact determined at surgery. Under the new system, the stage becomes susceptible to change based on secondary review or additional testing [39].

This hypothetical scenario illustrates the potential for significant diagnostic shifts: a tumor initially diagnosed as Stage IB Grade 2 endometrioid carcinoma could be reclassified as Clear Cell Carcinoma (Stage IIC) upon expert review, only to be ultimately reassigned to Stage IA mPOLEmut following the identification of a POLE mutation.

This instability creates profound confusion for patients and clinicians, complicates clinical trial enrollment (which often uses stage as a key inclusion criterion), and muddies cancer registry data essential for epidemiological tracking and research.

Additionally, the creation of Stage IIC as a “Bucket” for aggressive histotypes (Serous, Clear Cell, Carcinosarcoma, Grade 3 Endometrioid) is another critical point [5,6]. By grouping these diverse entities together, the system implies a biological and prognostic equivalence that robust clinical data contradict [5,6].

Lumping them into a single stage obscures these critical differences and removes the nuanced counseling necessary for personalized care. Additionally, for tumors placed in this “aggressive” bucket, the new system deprioritizes other powerful anatomical prognosticators like depth of myometrial invasion and cervical stromal involvement, which have historically been strong independent predictors of recurrence [40].

Moreover, the system amplifies the long-standing issue of Subjectivity in Lympho-vascular Space Invasion (LVSI) Quantification (Table 5) [5,6]. The 2023 system uses the distinction between “focal” and “substantial” LVSI to drive major upstaging decisions (e.g., from IA to IIB), considering as substantial the presence of multifocal or diffuse arrangement of LVSI or tumor cells in 5 or more lymph vascular spaces (Table 4) [5,6]. However, there is no globally standardized, reproducible definition of “substantial” LVSI [41,42]. Other authors have defined “substantial” LVSI (4 or more spaces) using data from the PORTEC-1 and -2 trials and the Danish Gynecologic Cancer Database (DGCD) collected in unstaged and under-staged cohorts of EC patients, after testing different lymphovascular space cutoffs associated with the risk of pelvic lymph node recurrence (PLNR) at 5 years. With a 5-year PLNR rate of 26%, the authors concluded that 4 or more spaces is the clinically meaningful cutoff for “substantial” LVSI [43,44].

In this way, while some guidelines suggest a cutoff involving multiple vessels or blocks, and although the prognostic role of substantial LVSI for recurrence, pelvic lymph node recurrence, overall and disease-specific survival independent of molecular subgroups and other clinicopathological features is undeniable, inter-observer variability among pathologists is high [5,6,41,42,43,44].

Technical artifacts, sampling density, possible mimickers (pseudo-emboli, MELF features), the impact of necrosis and pre-analytical phase issues, and the use of immunohistochemical stains further influence the count [41,42,43,44]. Basing a major treatment-escalating stage change on such a fragile and non-standardized parameter introduces significant risk of both overtreatment and undertreatment, undermining the goal of consistent care.

To resolve inter-observer variability, we propose the following standardization protocol:

(1) submit all tumor–myometrium interfaces (at least 1 block per cm of tumor diameter)
(2) systematically screen all H&E slides;
(3) exclude mimics (retraction artifacts, MELF pattern) using D2-40/CD31 IHC if equivocal;
(4) count LVSI-involved vessels on the single slide with the highest burden;
(5) classify as focal (1–3 vessels) or substantial (≥4 vessels) based on PORTEC/DGCD validation (5-year PLNR 26% vs. 6.7%);
(6) for borderline counts (3–4), assess pattern—diffuse/multifocal clusters may prompt upstaging;
(7) report uniformly as “LVSI: negative/focal (1–3)/substantial (≥4)” with pattern description.

To address this critical gap, we propose a pragmatic consensus pathway. First, adopt a validated numeric threshold: based on PORTEC and DGCD data, ≥4 LVSI-involved vessels on a single H&E slide consistently predicts pelvic lymph node recurrence (5-year risk 26% vs. 6.7% for 1–3 vessels), offering a reproducible alternative to the current ≥5 FIGO cutoff. Second, standardize the histopathological workflow: evaluate all available tumor-containing slides, use D2-40 or CD31 immunohistochemistry in equivocal cases, and apply a two-tiered reporting system (“focal” = 1–3 vessels; “substantial” = ≥4 vessels). Third, incorporate pattern-based criteria—diffuse, multifocal aggregates of tumor cells within vascular spaces—as a supplementary qualitative marker of high risk. While digital pathology and AI-assisted morphometric analysis represent the ultimate solution for objective quantification, their prospective clinical validation remains ongoing. Until then, this combined numeric–morphologic approach offers a feasible, evidence-based strategy to minimize inter-observer variability and ensure consistent staging.

Finally, the 2023 FIGO staging highlights the clinico-pathological distinctions of tumors with synchronous involvement of the uterine corpus and ovary into two categories (favorable and unfavorable outcome) [5,6]. A Stage IA3 category can be considered, an indolent behavior predicted, and a conservative management adopted when all the following criteria are met in a low-grade endometrial endometrioid carcinoma:

- endometrial and ovarian tumors are low grade
- no more than superficial myometrial invasion is present (<50% myometrial invasion)
- absence of extensive/substantial LVSI at any location
- no involvement of any other site (no additional metastases)
- the ovarian tumor is unilateral, limited to the ovary, without capsular invasion/breach/rupture

(pT1a)

Cases not fulfilling all the above criteria (high-grade tumors, over 50% myometrial invasion, substantial LVSI, bilateral ovarian involvement, capsular rupture, or with additional metastases) should be considered as extensive spread of the endometrial carcinoma to the ovary (stage IIIA1), more simply real metastasis, warranting the standard adjuvant treatment [5,6].

Since staging criteria are not infallible, molecular profiling is essential; according to FIGO 2023, certain cases could remain challenging in staging (low-grade EEC cases with not all fulfilled criteria of favorable outcome: 60% myometrial invasion, but unilateral ovarian involvement, absence of LVSI, absence of extrauterine or extraovarian diseases) [5,6]. These circumstances, as well as other pathological conditions (association with endometriosis and/or ovarian mullerian benign or borderline tumors), are still lacking unequivocal interpretation and should be considered worthy of a multidisciplinary approach, tumor- and patient-centered.

## 6. Refinements Within Stage IIIC to Reflect the Extent of Pelvic and Abdominal Lymph Node Metastases

The updated 2023 FIGO staging emphasizes the significance of metastatic extent in patients with advanced disease [5,6]. Differentiation of nodal involvement into macrometastases (size > 2 mm) and micrometastases (0.2–2 mm and/or number > 200 affected cells) in accordance with the approach adopted by the AJCC (American Joint Committee on Cancer) can significantly refine the prognosis [45,46].

Therefore, sentinel lymph node biopsy should be done for staging purposes in all patients with presumed uterus-confined disease. All sentinel lymph nodes should be subjected to pathological ultrastaging with a reasonable histological and immunohistochemical approach to combine cost-effectiveness and efficacy to detect low-volume metastatic disease. Both macrometastases (pelvic IIIC1ii or para-aortic IIIC2ii)—and micrometastases (pN1[mi]—pelvic IIIC1i or para-aortic IIIC2i)—are regarded as a metastatic involvement [45,46]. This substaging is based on the observed better prognosis in patients with nodal micrometastasis. On the other hand, SLN macrometastases (both in the pelvis and para-aortic areas) are independently linked to extensive lymphatic dissemination risk, non-SLN dissemination, and distant recurrences, although the risk of non-vaginal recurrences is debated [5,6,45,46].

Further refinements could be oriented to define the association between location (intracapsular vs. extracapsular extension) of lymphatic metastasis and outcomes.

This suggests the importance of including not only SLN metastasis size but also location of metastatic deposits and measurements of extracapsular extension in positive SLNs within the pathological report, as all these features may influence both the locoregional therapeutic strategy and lymph nodal surgical management [47]. The identification of pathological characteristics within positive SLNs, coupled with uterine pathological risk factors (aggressive histologies, LVSI, histological grade), might enhance our ability in the recurrence risk stratification and customization of appropriate adjuvant treatment.

Finally, the prognostic significance of isolated tumor cells (deposits ≤ 0.2 mm and up to 200 cells; pN0[i+]) is unclear; therefore, to date, their identification does not upstage a carcinoma [5,6,7,8]. Prospective larger studies are warranted to evaluate the clinical significance and long-term outcomes of patients with isolated tumor cells, in conjunction with the tumor molecular profile

## 7. Staging Versus Risk Stratification

Discrepancies between the 2023 FIGO system and ESGO/ESTRO/ESP guidelines highlight a central debate: the formal integration of molecular biology into staging versus its application as a secondary modifier in risk assessment [5,6]. Anatomical staging, as historically conceived by FIGO, is designed to be a static, descriptive language of tumor burden (Tumor, Node, Metastasis). Its primary virtues are reliability, reproducibility, and stability over time [47]. Integrating evolving molecular data into the 2023 staging system makes the framework unstable. Frequent updates required for new biomarkers such as L1CAM, *CTNNB1*, or SWI/SNF may lead to clinical confusion and rapid obsolescence [5,6]. Integrated Risk Stratification, as suggested by the European guidelines, offers a more flexible and adaptive model [7,8]. Here, the anatomical stage remains an immutable foundation based on surgical-pathological findings. Molecular and refined clinicopathological features are then layered onto this foundation to assign patients to “risk groups” (e.g., low, intermediate, high-intermediate, high) [7,8]. This approach enables personalized decision-making without destabilizing the core language of staging. For example, a patient with anatomically Stage IA disease that is p53-abnormal would be classified as “High Risk,” triggering consideration of adjuvant therapy. Conversely, a patient with anatomically Stage II disease that is POLE-mutated could be considered “Low Risk,” justifying therapy de-escalation.

The proposed solution to mitigate the current challenges is a pragmatic decoupling of these two concepts. Staging should revert to or remain a “classic” anatomical system, ensuring it can be applied universally and reproducibly in any clinical setting worldwide, preserving global comparability. Risk stratification should be an evolving, integrated algorithm that overlays all available data (molecular, histological, pathological) onto the anatomical stage to guide therapy. This dual-track system ensures that the essential, universal language of cancer (stage) is not held hostage to resource availability, while still enabling the most sophisticated, personalized treatment planning where advanced diagnostics are accessible.

### Proposed Clinical Decision-Making Model

**Figure 1:**
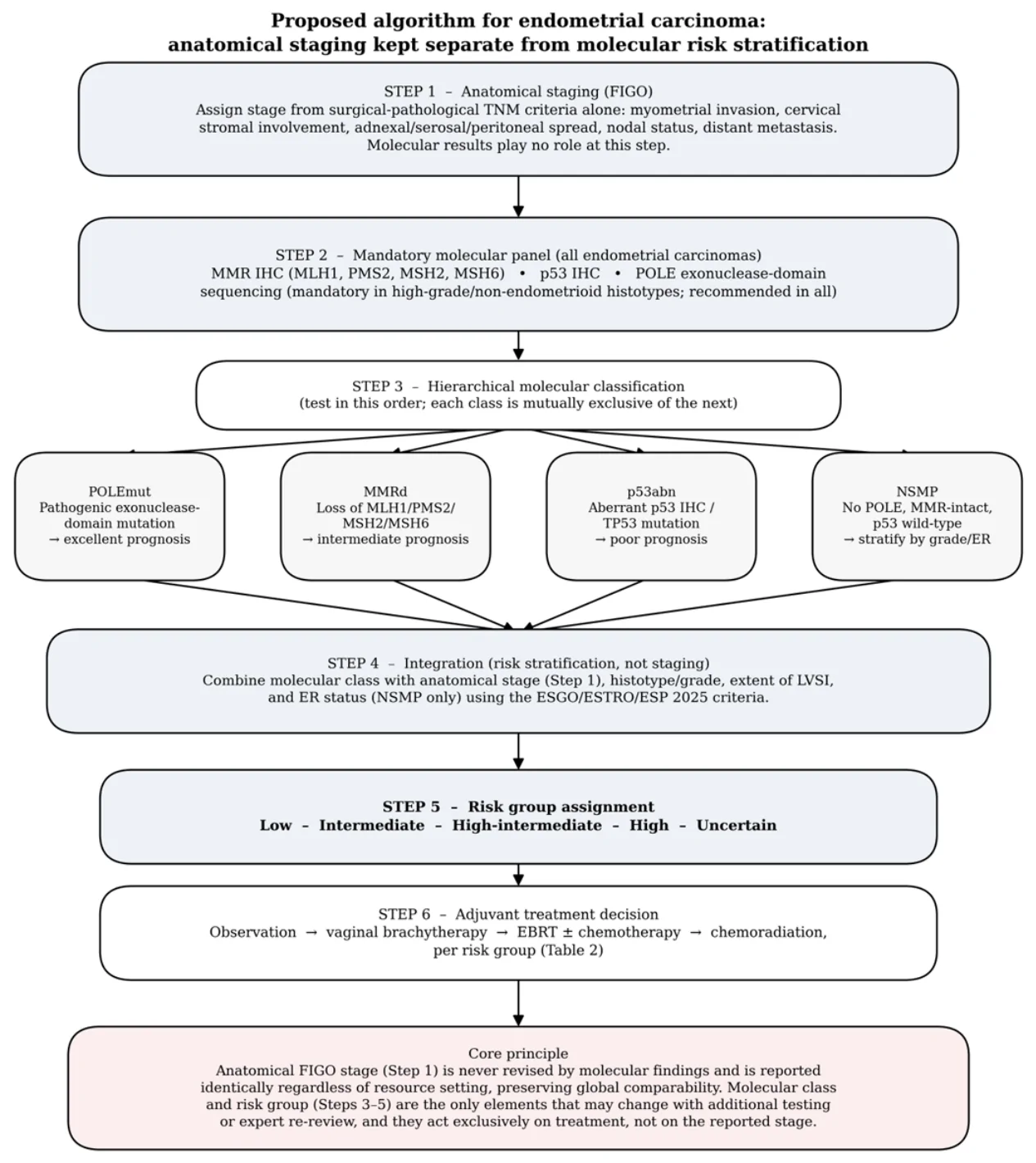
A pragmatic implementation of a sequential histo-molecular approach: first the FIGO stage and the pathological diagnosis to assess tumoral burden and spread; second the molecular testing to classify the tumor genetically; third the integrated step to define risk categories and guide therapies.

A pragmatic implementation of this dual-track approach could follow a sequential model. First, the anatomical FIGO stage would be established independently on the basis of surgical and pathological findings (Figure 1). Second, molecular testing would classify the tumor into POLE-mutant, MMR-deficient, p53-abnormal, or NSMP categories. Third, molecular and pathological features (including histotype, grade, myometrial invasion, LVSI, and molecular subgroup) would be integrated to determine the patient’s prognostic risk group and guide adjuvant treatment. Importantly, molecular information would complement rather than retrospectively alter the anatomical stage. In cases of discordant or uncertain findings, multidisciplinary review and expert pathological assessment would be recommended. This dual-track model would preserve a stable and internationally comparable anatomical stage while allowing molecular and pathological characteristics to drive individualized treatment decisions.

Figure 1 summarizes this proposal as a stepwise algorithm.

## 8. Quantifying the Global Accessibility Gap

The ethical concern regarding differential access to molecular testing is not merely theoretical. Recent multinational surveys and implementation studies have documented the disparity in molecular diagnostic capabilities across regions [48].

A cross-sectional survey of 17 Eastern European countries conducted between December 2024 and September 2025 found that full molecular profiling (p53, POLE, and MMR) was available in only 29.4% of countries, with partial access in 70.6% and complete absence in a substantial proportion [49]. Lack of reimbursement and accredited laboratories were identified as the primary barriers. At the institutional level, testing was routine in 41.0% of centers, selective in 27.9%, and completely unavailable in 31.1% [49]. Importantly, comprehensive classification was significantly more available in settings with national/public reimbursement (79.2% vs. 18.9%, *p* < 0.0001), underscoring that access is largely determined by healthcare financing rather than clinical need [49].

The situation is even more critical in low- and middle-income countries (LMICs). A commentary on FIGO 2023 implementation in LMICs reported that fewer than 40% of centers have access to immunohistochemistry (the most basic platform for MMR and p53 assessment) and there are fewer than three pathologists per million population in many regions of Africa [50]. The high expense of molecular testing renders it inaccessible for many institutions, threatening to exacerbate existing global disparities in gynecologic cancer care. In one LMIC center, only 2 of 32 patients eligible for molecular classification could afford POLE testing [50]. This is particularly concerning given that endometrial cancer–related disability-adjusted life years in sub-Saharan Africa rose by 75% between 1990 and 2017, with incidence and mortality projected to double by 2040 [50].

The consequences extend beyond diagnosis to clinical outcomes. A prospective real-world study from India demonstrated that molecular classification changed multidisciplinary treatment recommendations in 20.5% of early-stage cases (escalation in 12.3%, de-escalation in 8.2%), with therapy modification most frequent in p53-abnormal tumors (62.5%) [51]. Without molecular testing, these patients would receive either inadequate or excessive treatment—a direct consequence of diagnostic unavailability.

## 9. Conclusions and Future Directions

The integration of molecular classification into endometrial cancer management represents one of the most important advances in gynecologic oncology over the last decade. While the 2023 FIGO staging system successfully acknowledges tumor biology, several practical issues (unequal access to molecular testing, stage instability, and the evolving landscape of predictive biomarkers) highlight the need for continued refinement.

Future studies should focus on harmonizing definitions, expanding access to essential molecular testing in resource-limited settings, and refining the integration of emerging biomarkers into clinical practice. The future evolution of endometrial cancer classification will depend on these essential steps, ensuring that staging remains a stable, universal language while risk stratification becomes increasingly precise and personalized.

## Figures and Tables

**Table 1:** Molecular classification of endometrial carcinoma.

Molecular Group	Frequency	Key Alterations	Prognosis	Therapeutic Implications
POLEmut	7–10%	POLE exonuclease domain mutations	Excellent	De-escalation of therapy
MMRd	25–30%	Loss of MLH1/MSH2/MSH6/PMS2	Intermediate	Immune checkpoint inhibitors
p53abn	~25%	TP53 mutations, CNH profile	Poor	Aggressive treatment, potential PARPi
NSMP	~40%	No specific alteration	Variable	Relies on clinicopathological factors

**Table 2:** ESGO/ESTRO/ESP 2025 risk stratification.

Risk Group	Criteria (Simplified)	Recommended Treatment
Low	Early stage + favorable molecular profile	No adjuvant therapy
Intermediate	Limited invasion + MMRd/NSMP low-grade	±vaginal brachytherapy
High-intermediate	Deeper invasion or LVSI	EBRT ± chemotherapy
High	p53abn or advanced stage	Chemoradiation
Uncertain	Selected early/advanced POLE or NSMP high-grade	MDT-based decision

**Table 3:** Histotype vs. molecular profile.

Histotype	Typical Molecular Group	Exceptions	Clinical Implication
Endometrioid G3	Mixed	POLE/MMRd	Avoid overtreatment
Serous	p53abn	POLE/MMRd	Mandatory molecular testing
Clear cell	NSMP/MMRd	Rare p53	Intermediate behavior
Carcinosarcoma	p53abn	MMRd/POLE	Heterogeneous
UEC/DEC	MMRd	SWI/SNF-deficient	Highly aggressive subset

**Table 4:** Clinical algorithm for rare endometrial histotypes.

Step	Action	Key Points
**1**	Anatomical stage	Standard surgical-pathological criteria (fixed, independent of histotype).
**2**	Molecular testing	POLE, MMR, p53; plus KRAS (MLEC), HER2 (GTEC), RB1 (NEEC).
**3**	Molecular class	MLEC → NSMP (KRAS+); GTEC → p53abn/NSMP; NEEC → variable.
**4**	Histotype risk	MLEC, GTEC, small cell NEEC → High risk (regardless of stage).
**5**	Dual-track assignment	Anatomical stage (e.g., IA) + Histotype risk + Molecular data → Guide treatment.
**6**	Multidisciplinary review	Mandatory tumor board discussion for all rare histotypes.
**7**	Targeted therapy	MLEC: MEK inhibitors; GTEC: anti-HER2 (if ERBB2+); NEEC: PARPi (if HRD+) or ICIs (if MMRd).

**Table 5:** LVSI definitions and clinical impact.

**Absent LVSI**
Definition: No tumor cells in vascular spaces
Source: PORTEC-1/2; Peters et al.
Implication: Lowest recurrence risk
Outcome: 5-year pelvic nodal recurrence 3.3%
**Focal LVSI**
Definition: 1–3 vessels on most affected slide
Source: PORTEC-1/2; DGCD; Peters et al.
Implication: Limited increase in recurrence risk
Outcome: 5-year pelvic nodal recurrence 6.7%
**Substantial LVSI (PORTEC-1/2)**
Definition: ≥4 vessels on most affected slide
Source: PORTEC-1/2; DGCD; Peters et al.
Implication: Strong adverse prognostic factor; upstaging to Stage IIB
Outcome: 5-year pelvic nodal recurrence 26.3%
**Substantial LVSI (FIGO/WHO)**
Definition: ≥5 vessels
Source: WHO 2020; FIGO 2023
Implication: May upstage to Stage IIB
Outcome: 5-year PFS 68.7%; aHR 1.18 (95% CI 1.00–1.39)
**Diffuse/Pattern-based LVSI**
Definition: Diffuse/multifocal clusters; no numeric threshold
Source: Restaino et al.
Implication: Adverse prognostic feature, especially borderline counts
Outcome: Nodal metastasis 33%; distant recurrence 24.9%

## Data Availability

No new data were created or analyzed in this study. Data sharing is not applicable to this article.

## Abbreviations

The following abbreviations are used in this manuscript:

FIGO	International Federation of Gynecology and Obstetrics
POLE	DNA Polymerase Epsilon
LVSI	Lymphovascular Space Invasion
MELF	Microcystic, Elongated, and Fragmented (pattern)
PLNR	Pelvic Lymph Node Recurrence
PORTEC	Post Operative Radiation Therapy in Endometrial Cancer
DGCD	Danish Gynecologic Cancer Database
SLN	Sentinel Lymph Node
AJCC	American Joint Committee on Cancer
pN	Pathological Node (stage)
ESGO	European Society of Gynaecological Oncology
ESTRO	European Society for Radiotherapy and Oncology
ESP	European Society of Pathology
L1CAM	L1 Cell Adhesion Molecule
CTNNB1	Catenin Beta 1
SWI/SNF	SWItch/Sucrose Non-Fermentable
AI	Artificial Intelligence
TCGA	The Cancer Genome Atlas
NSMP	No Specific Molecular Profile
MMR	Mismatch Repair
MMRd	Mismatch Repair Deficient
p53abn	p53 Abnormal
HER2	Human Epidermal Growth Factor Receptor 2
ARID1A	AT-Rich Interaction Domain Containing Protein 1A
BRG1	Brahma-Related Gene 1
INI1	Integrase Interactor 1
EMCG	Endometrial Carcinoma of Gastrointestinal-type
WHO	World Health Organization
